# Curcumin alters distinct molecular pathways in breast cancer subtypes revealed by integrated miRNA/mRNA expression analysis

**DOI:** 10.1002/cnr2.1596

**Published:** 2022-01-04

**Authors:** Snehal Nirgude, Sagar Desai, Bibha Choudhary

**Affiliations:** ^1^ Institute of Bioinformatics and Applied Biotechnology Bangalore India; ^2^ Division of Human Genetics Children's Hospital of Philadelphia Philadelphia USA; ^3^ Manipal Academy of Higher Education Manipal India

**Keywords:** breast cancer, curcumin, pathways, transcriptomics

## Abstract

**Background:**

Curcumin is well known for its anticancer properties. Its cytotoxic activity has been documented in several cancer cell lines, including breast cancer. The pleiotropic activity of curcumin as an antioxidant, an antiangiogenic, antiproliferative, and pro‐apoptotic, is due to its diverse targets, such as signaling pathways, protein/enzyme, or noncoding gene.

**Aim:**

This study aimed to identify key miRNAs and mRNAs induced by curcumin in breast cancer cells MCF7, T47D (hormone positive), versus MDA‐MB231 (hormone negative) using comparative analysis of global gene expression profiles.

**Methods:**

RNA was isolated and subjected to mRNA and miRNA library sequencing to study the global gene expression profile of curcumin‐treated breast cancer cells. The differential expression of gene and miRNA was performed using the DESeq R package. The enriched pathways were studied using cluster profileR, and integrated miRNA–mRNA analysis was carried out using miRtarvis and miRmapper tools.

**Results:**

Curcumin treatment led to upregulation of 59% TSGs in MCF7, 21% in MDA‐MB‐231 cells, and 36% TSGs in T47D, and downregulation of 57% oncogenes in MCF7, 76% in MDA‐MB‐231, and 91% in T47D. Similarly, curcumin treatment led to upregulation of 32% TSmiRs in MCF7, 37.5% in MDA‐MB231, and 62.5% in T47D, and downregulation of 77% oncomiRs in MCF7, 50% in MDA‐MB231 and 28.6% in T47D. Integrated analysis of miRNA–mRNA led to the identification of a common NFKB pathway altered by curcumin in all three cell lines. Analysis of uniquely enriched pathway revealed non‐integrin membrane–ECM interactions and laminin interactions in MCF7; extracellular matrix organization and degradation in MDA‐MB‐231 and cell cycle arrest and G2/M transition in T47D.

**Conclusion:**

Curcumin regulates miRNA and mRNA in a cell type‐specific manner. The integrative analysis led to the detection of miRNAs and mRNAs pairs, which can be used as biomarkers associated with carcinogenesis, diagnostic, and treatment response in breast cancer.

## 1 INTRODUCTION

Molecular tumor heterogeneity is the biggest hurdle in breast cancer treatment.^1^, ^2^, ^3^ Though surgery, radiation therapy, hormonal therapy, immunotherapy, and targeted therapy are routine treatment regimes, breast cancer metastasis, drug resistance, and relapse lead to poor patient survival.^4^, ^5^ Hence the development of newer and better therapeutics is required. Drug development processes can be challenging due to the complexity of the biological system.^6^ Therefore exploring bioactive substances from herbs, medicinal plants, and natural products can be utilized as these have the potential to minimize the side effects and resistance associated with chemotherapy.

Curcumin, a polyphenol extracted from turmeric, is well known for its multifaceted properties like anti‐inflammatory, antioxidant, anti‐bacterial, anti‐malarial, and anticancer.^7^, ^8^, ^9^ Clinical and preclinical studies have validated the role of curcumin in varied human chronic diseases, including cancer.^10^ The therapeutic potential of curcumin can be attributed to its capability to regulate both epigenome and transcriptome.^10^, ^11^

Out of 25 000 genes (encoded by the human genome), roughly 600 targets of the drug, primarily receptors, and enzymes, have been targeted in the diseased state.^12^ With advances in next‐generation sequencing technology (NGS), the time required for drug target study and biomarker discovery has reduced significantly.^6^ RNA‐seq is an important application of (NGS) because it can generate comprehensive transcriptome information at different levels, including quantification of protein‐coding and noncoding gene expression, identification of noncoding RNAs (ncRNAs), and/or fusion genes, and determination of alternative splicing. Using RNA‐seq to catalog events in drug‐treated samples against normal provides insights into pathway analysis, gene ontology, and gene regulation.^13^ RNA‐seq analysis helps in the unbiased detection of both coding and noncoding novel transcripts and transcripts with low abundance.^14^ As compared to microarrays, RNA‐seq is more sensitive and accurate in discriminating between highly similar sequences.^15^ Tools like the NCI Transcriptional Pharmacodynamics Workbench (NCI TPW) have been developed to decipher gene expression, molecular pathways, drug target, and drug sensitivity across the NCI‐60 panel in response to 15 anticancer agents.^16^

Since drugs cannot target every protein, alternate such as miRNA therapeutics can be utilized. MiRNAs are ~22 nucleotides noncoding RNAs that regulate gene expression by RNA interference post‐transcriptionally.^17^, ^18^ These noncoding RNAs play an important role in protein expression by modifying the sequence structure of expression of the mRNA.^17^ This regulation is based on the complementarity of miRNA with the 3'UTR region of target mRNA. However, miRNAs can also bind to the 5'UTR or coding region of target mRNA and activate translation.^19^ miRNAs can modulate epigenetic machinery, and reciprocally, their expression can be modulated by the epigenetic machinery.^18^ miRNA‐seq gives the miRNA profile of the disease and the miRNA profile induced by the drug, which can target undruggable proteins.^20^

Integrated RNA‐seq and miRNA‐seq analysis provide insight into drug‐induced global alterations in the transcriptome. It also helps in deciphering the molecular mechanism of the disease via gene expression and gene regulation.^21^ Interestingly, genes and miRNAs involved in drug resistance can also be studied by comparing expression profiles of resistant and non‐resistant cells using the whole transcriptome approach.^21^ Thus, genome‐wide transcriptome profiling is instrumental in accurately representing the expression pattern of the coding and noncoding genome in homeostasis and early and late events leading to disease.^22^

RNA‐seq has been employed to study the changes in tumorigenesis in the presence of different drugs such as curcumin,^23^ shikonin,^14^ gefitinib,^24^ Gallic acid,^25^ benzo[α]pyrene (BaP),^26^ Fructus Meliae Toosendan (FMT),^27^ citral,^28^ Quercetin^29^ and so on. In this study, we have explored the whole transcriptome (mRNA and miRNA) effect post‐curcumin treatment in MDA‐MB‐231, MCF7, and T47D cells. The analysis had led to the identification of drug‐induced cell specific miRNA–mRNA networks, pathways, and new targets involved in three different breast cancer cells.

## 2 MATERIALS AND METHODS

### 2.1 Cell culture

MDA‐MB‐231, MCF7, and T47D cells were purchased from the National Centre of Cell Culture (NCCS), Pune, Maharashtra, India. All the cell lines were authenticated at NCCS by short tandem repeat (STR) analysis. Dulbecco's Modified Eagles Medium (DMEM high glucose with L‐glutamine; Lonza) was used for MDA‐MB‐231 cells, Eagle's Minimum Essential Medium (EMEM; Lonza supplemented with non‐essential amino acids [NEAA] from MP biomedicals) was used for MCF7 cells, and Roswell Park Memorial Institute‐1640 (RPMI; Lonza) media was used for T47D cells. All three media were supplemented with heat‐inactivated 10% fetal bovine serum (Gibco), 100 IU mg/ml penicillin/streptomycin (Gibco) at 37°C in a humidified atmosphere containing 5% CO_2_. 100 mM curcumin stocks were prepared in DMSO, and the treatment was given so that all treatments had equal concentrations of dimethyl sulfoxide (DMSO) between 0.1% and 0.2%. The molecular signatures of each cell line are tabulated in Table 1.

**TABLE 1:** Characteristic Molecular Signatures of Cell lines used in the study

Characteristics	MCF7	T47D	MDA‐MB‐231
Breast Cancer Subtype^30^, ^31^	Luminal A	Luminal A	Claudin‐low triple negative
ER,PR, Her2 status^30^, ^31^	ER +ve, PR +ve, Her2 −ve	ER +ve, PR +ve, Her2 −ve	ER −ve, PR −ve, Her2 −ve
p53 Status^32^	Wild type	Mutant (protein variant p.L194F)	Mutant (protein variant p.R280K)
BRCA1 status^33^	Wild type	Wild type	Wild type

### 2.2 RNA preparation and HiSeq2500 sequencing

#### 2.2.1 Drug treatment, RNA isolation, and library preparation

Drug treatment and RNA isolation: 0.75 × 10^5^ cells were seeded in each well of a 6‐well plate. After 24 h, MCF7, T47D, and MDA‐MB‐231 cells were treated with 10 μM curcumin. After 48 h of treatment, cells were trypsinized, and cells from three wells having the same treatment were pooled together. After two PBS washes, RNA extraction was done using Trizol Reagent (Ambion) following the manufacturer's recommendations. RNA concentration and purity were checked using Qubit (Invitrogen, Life Technologies), and its integrity was examined by capillary electrophoresis (Tapestation, Agilent Technologies) to ensure RNA integrity number >9, for a good RNA library preparation. Paired‐end RNA‐seq libraries were prepared using Illumina TruSeq RNA Library Prep Kit v2.

mRNA library preparation: In brief, from the total RNA, mRNAs were separated using oligo‐dT beads and fragmented to 200–250 bp. After cDNA was synthesized, the ends were repaired for blunt ends, and the 3' ends were adenylated. To the adenylated sites, adapters were linked, and subsequently, PCR amplification of the library was done. After constructing the libraries, their concentrations and insert sizes were detected using Qubit and Agilent Tapestation, respectively. High throughput sequencing was performed using Illumina HiSeq2500 to obtain 100‐bp paired‐end reads.

#### 2.2.2 miRNA library preparation

RNA isolation was done as mentioned above, and RNA sample was given for library preparation. miRNA‐library preparation was outsourced to SciGenom Labs, India. In brief, after checking the quality, RIN of RNA, 3′ and 5′ adapters were ligated to the short mature miRNA sequences. After adapter ligation, reverse transcription was done to obtain single‐stranded cDNAs. The cDNA was then PCR amplified, and the amplicons were run on 8% native PAGE. The ~150 bp library was gel purified and the quality of libraries was checked using Tapestation 2200, Agilent. The libraries were pooled and sequenced using Hiseq 2500, Illumina.

### 2.3 Differential expression analysis

#### 2.3.1 mRNA‐seq

Data analysis was carried out, beginning with filtering raw reads output from Illumina Hiseq2500 platform. The sequencing depth for each sample was >40 million reads. The quality of the reads was checked using the FastQC tool.^34^ The reads were aligned with Bowtie2^35^ to the hg38 reference genome. The tool coverage bed from BEDTools^36^ was used to extract the count per transcript per sample using the annotation files. Differential expression analyses of drug‐treated samples against control samples were performed using the DESeq R package.^37^ Heatmap and hierarchical clustering were done to understand the expression profile based on the value of significantly differentially expressed transcripts.

#### 2.3.2 miRNA‐seq

Data analysis was carried out in the following steps: filtering was done on raw reads output from Illumina Hiseq2500 platform. The sequencing depth for each sample was >10 million reads. The quality of the reads was checked using the FastQC tool,^34^ and >90% of reads had a Phred score (*Q*) >30. Trimming was done using trim_galore^38^ to obtain read lengths of 18–25 bp. The alignment was performed using Bowtie2,^35^ and differentially expressed miRNA was obtained, as mentioned above.

### 2.4 Enrichment and pathway analysis for mRNA‐seq data

For pathway analysis of the differentially expressed genes, we have used clusterprofileR.^39^ The significant pathways were considered based on *p*‐value.

### 2.5 Integrated enrichment and network analysis of mRNA–miRNA

miRtarvis+^40^, ^41^ and miRmapper^42^ tools were used for studying^40^, ^41^ the interaction between mRNA and miRNA. The mRNA–miRNA network was generated using miRtarvis+.^40^, ^41^ Every miRNA target was verified using TargetScan,^43^ mirTarBase^44^ and miRDB.^45^

### 2.6 Quantitative real‐time PCR

cDNA was synthesized from 1–2 μg RNA using cDNA synthesis kit from Takara Bio according to the manufacturer's instructions. The PCR reaction was performed using StepOnePlus™ real‐time PCR system from Applied Biosystems using iTaq™ Universal SYBR® Green supermix from Bio‐Rad with PCR primers for genes (ANKRD12, CTDSP1, ZNF292, FAM83D) and miRNAs (miR‐16, miR‐34). The sequences for the primers are provided in Supplementary Table 4. The relative level of the target gene from each sample was determined by normalizing it to β‐actin. All experiments were done in triplicates and repeated at least twice to duplicate results.

### 2.7 Immunoblotting

To perform this assay, 0.8 × 10^5^ MCF7 cells/ml were seeded and treated with curcumin (1, 5, and 10 μM) for 48 h, and whole cell lysate was prepared as described in.^46^, ^47^ 30 μg of cell lysates were electrophoresed on 10%–12% of SDS‐polyacrylamide gel electrophoresis (PAGE) and transferred to Polyvinylidene fluoride membrane. The membrane was blocked using 5% skim milk in 1× PBS and then probed with primary antibodies: GAPDH were purchased from Cloud clone Corp., NF‐KB from Biolegend, Horseradish peroxidase‐labeled secondary anti‐rabbit antibody from Cell Signalling Technology. The membrane was probed with appropriate antibodies and was developed using a chemiluminescence reagent (Clarity Western ECL blotting substrateBiorad). The blot image was captured by using the Chemidoc‐XRS Biorad gel doc system, and the protein band images were quantified using GelQuant.Net, BiochemLab solutions.

## 3 RESULTS

### 3.1 Transcriptomic analysis of breast cancer cells upon curcumin treatment using RNA‐seq

The dose–response study of curcumin in breast cancer cells (MCF7, MDA‐MB‐231, T47D) was performed,^47^ and accordingly, the concentration of curcumin was determined. Differential gene expression analysis was performed after 48 h curcumin (10 μM) treatment on three breast cancer cells of different origins MCF‐7 (luminal), T47D (luminal), and MDA‐MB‐231 (TNBC). The data was generated by pooling three biological replicates, and gene expression profiling was performed using RNA‐seq. On average, 40 million reads were generated, with ~80%–84% alignment with the hg38 reference genome for all the RNA‐seq data (Supplementary Table 1).

The PCA plots of differentially expressed genes among three breast cancer cell lines MCF7, MDA‐MB231, and T47D, treated with curcumin (Supplementary Figure 1), show that the three cell lines clustered away from each other suggesting differences in the origin of cell lines and their expression patterns.

**FIGURE 1:**
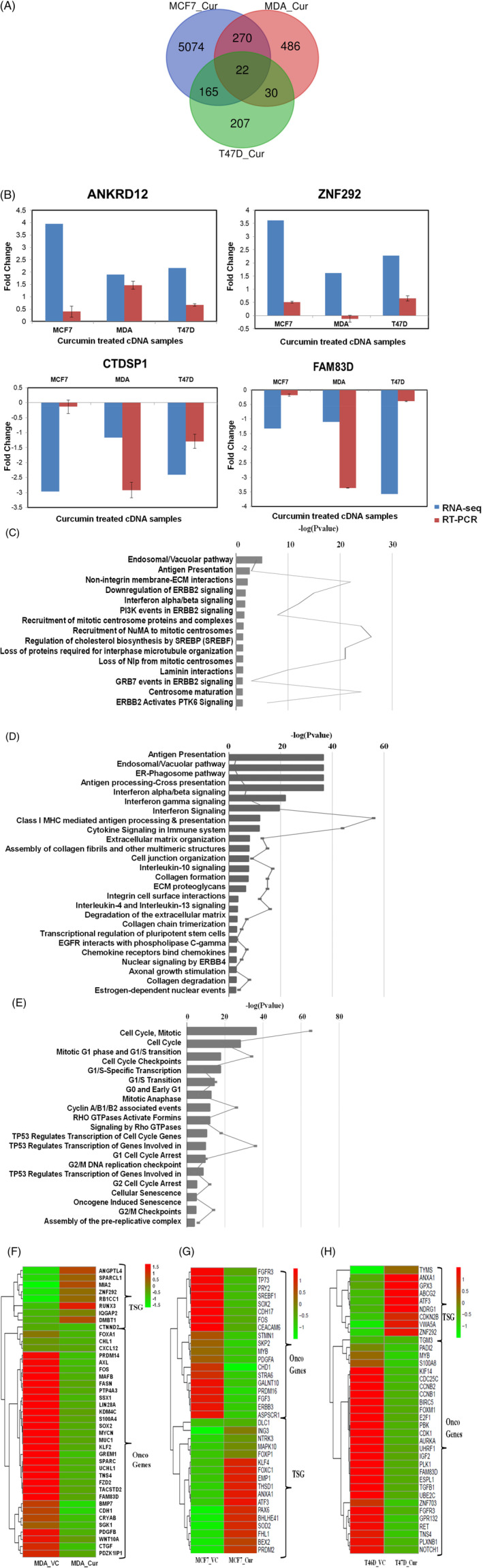
Venn diagram for DE genes from curcumin treated MCF7, MDA‐MB‐231, and T47D cells (A). (B) Real‐time PCR validation of common genes. Pathways regulated by curcumin in MCF7 (C), MDA‐MB‐231 (D), and T47D (E). Bars represent *p*‐value for the pathway and the trendline represents the number of DE genes in the pathway. Heatmap showing DE of TSGs and oncogenes in MDA‐MB‐231 (F), MCF7 (G), and T47D (H) cells upon curcumin treatment

A total of 5530 genes in MCF7, 807 in MDA‐MB‐231423 in T47D genes were differentially expressed (DE) (Log2 Fold Change > ±1, *p*‐value <.05). A transcriptome summary shows the percentage of up and down‐regulated genes among the total DE genes (Supplementary Figure 2A). 45%, 32%, 21% DE genes were upregulated, and 55%, 68%, 79% DE genes were downregulated in MCF7, MDA‐MB‐231, and T47D respectively. 22 DE genes were common to all three cell lines after curcumin treatment (Figure 1A). 5074 DE genes were unique to MCF7, 486 were unique to MDA‐MB‐231, and 207 were unique to T47D (Figure 1A). 4.6% DE genes were common between MCF7 and MDA‐MB‐231, 3% DE genes between MCF7 and T47D, whereas 4.1% DE genes were common between T47D and MDA‐MB‐231. 91.7%, 60%, 48.9% DE genes were uniquely altered in MCF7, MDA‐MB‐231, and T47D cells after curcumin treatment. Among the 22 common genes, 15 DE genes were downregulated, and 3 DE genes were upregulated in all three cell lines upon curcumin treatment. Oncogenic genes like FAM83D, CTDSP1, and SAPCD2 were downregulated in all three cells, and tumor suppressor genes (TSGs) like ZNF292, ANKRD12, NKAPL, and CCL21 were upregulated in all three breast cancer cells upon curcumin treatment. FAM83D^48^ and SAPCD2^49^ are known to promote cell proliferation, induce cell motility, and hence their downregulation by curcumin is a positive outcome.

We validated few genes like ZNF292, ANKRD12, CTDSP1, and FAM83D by real‐time (RT) PCR. The qRT‐PCR results showed a similar differential expression pattern, validated the expression of genes, that is upregulation/ downregulation, as obtained from the RNA‐Seq results. However, the log_2_ fold change of the differentially expressed genes in the qRT‐PCR did not perfectly match the RNA‐Seq. For example, the ANKRD12 gene was upregulated by 3.95,1.89, and 2.16 log_2_ fold change in RNA‐seq data of curcumin treated MCF7, MDA‐MB‐231, and T47D cell respectively. In contrast, in RT‐PCR, the ANKRD12 gene was upregulated by 0.39, 1.46, and 0.67 log_2_ fold change for curcumin treated MCF7, MDA‐MB‐231, and T47D.

Further, DE genes were subjected to pathway analysis in all three cell lines to identify altered pathways upon curcumin treatment. Common pathways altered in MCF7, MDA‐MB‐231 by curcumin were ER‐Phagosome pathway, Antigen processing‐Cross presentation Interferon gamma signaling, Endosomal/Vacuolar pathway, Antigen Presentation: Folding, assembly and peptide loading of class I MHC pathways. Extracellular matrix (ECM) related interactions with non‐integrin membrane and laminin pathways were regulated by curcumin uniquely in MCF7(Figure 1C) cells, whereas ECM degradation and Collagen degradation were enriched in MDA‐MB‐231 (Figure 1D). Pathways related to the cell cycle were uniquely regulated in T47D cells by curcumin (Figure 1E).

Cancer is driven by an imbalance of oncogene and tumor suppressor gene (TSG) expression. Thus, we analyzed the significant DE genes for TSGs and oncogenes. For TSGs, we used the TSG database^50^ and selected TSGs associated with breast invasive carcinoma samples, and for oncogenes, we used the Oncogene database.^51^ 589 TSGs were found in the TSG database for breast adenocarcinoma (BRCA) and 803 oncogenes in the Oncogene database. The percentage of upregulated TSGs and downregulated oncogenes were calculated using the significant DE genes after curcumin treatment. 59% TSGs were upregulated in MCF7, 21% in MDA‐MB‐231 cells and 36% TSGs were upregulated in T47D after curcumin treatment. 57% oncogenes were downregulated in MCF7, 76% in MDA‐MB‐231, and 91% in T47D cells after curcumin treatment. Heatmaps were plotted for these TSGs and oncogenes, indicating cell type specific regulation of TSGs after curcumin treatment in all three breast cancer cells namely, MDA‐MB‐231, MCF7, and T47D (Figure 1F‐H).

### 3.2 Comparative analysis of curcumin treatment in breast cancer cell line at a chromosomal level

**FIGURE 2:**
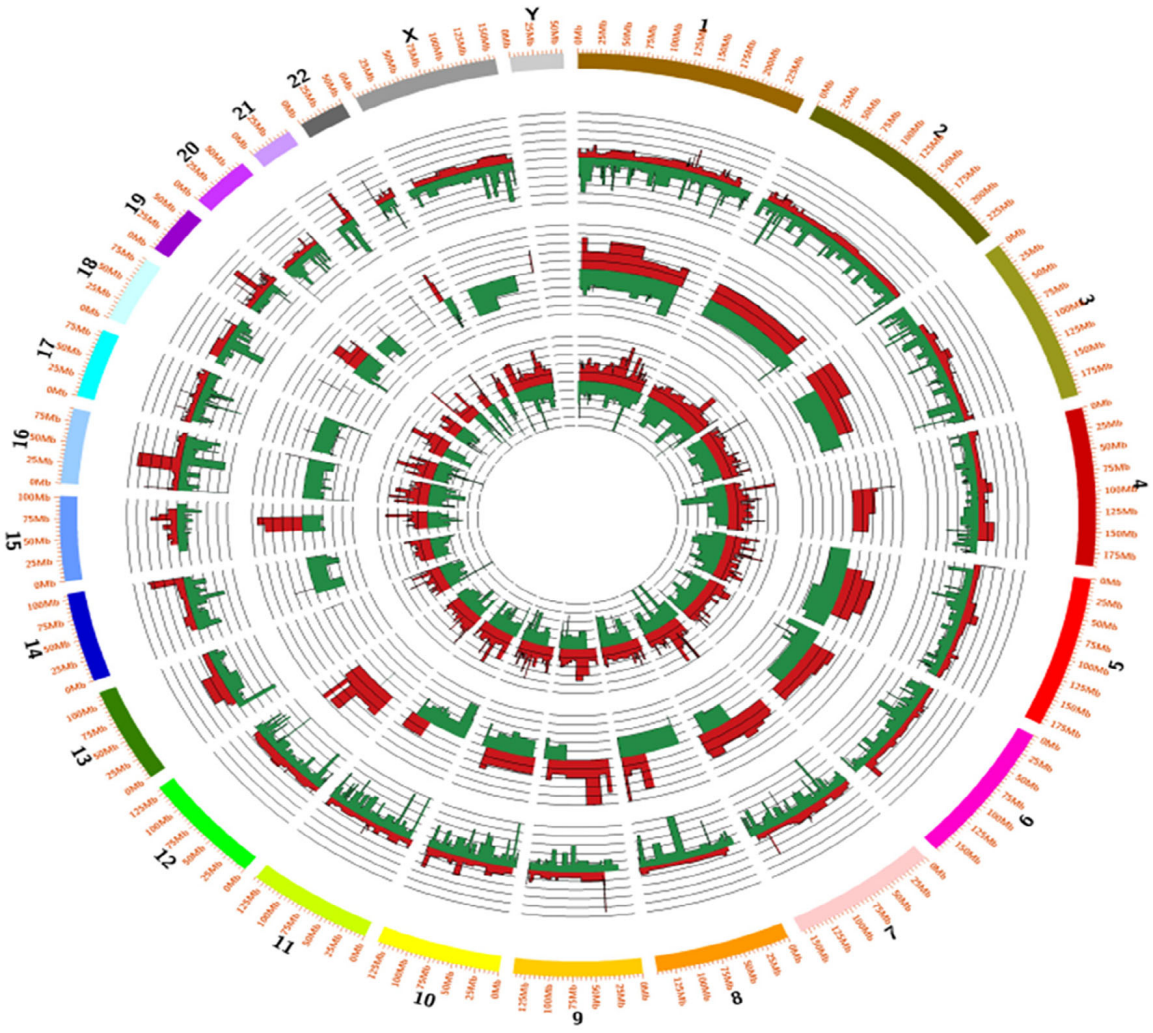
Circos plot for curcumin treatment in MCF7, MDA‐MB‐231, and T47D cells (Innermost Track: MCF7, Middle track: T47D, Outermost Track: MDA‐MB‐231; Red color highlights upregulation, and green color highlights downregulation)

The chromosomal distribution of the altered transcripts (significant DE genes) across all cell lines was plotted using Circos.^52^ As expected, Chromosome Y showed no expression of genes. Curcumin showed an even distribution of down and upregulated genes in MCF7 and MDA‐MB‐231 cell lines at the chromosomal level. However, curcumin‐treated T47D showed an absence of gene expression from chromosomes 13, 18, and 21; genes from chromosomes 14, 16, 17, 20, and X were downregulated, and genes from chromosomes 4 and 12 were upregulated (Figure 2). Chromosome 13, 16, 17, and 18 are associated with breast cancer.^53^, ^54^, ^55^ For example, BRCA1 and BRCA2, two major genes mapped to the long arms of chromosomes 17 and 13, determine predisposition to breast cancer.^53^ Loss of heterozygosity (LOH) at the long arm of chromosome 16 is a genetic alteration that is frequently observed in differentiated ductal breast cancer.^54^ This analysis, combined with all earlier analyses, validates the observation that the transcriptome's global profiling induced by the drug is cell line specific. Thus, curcumin altered genes in a unique fashion specific to each breast cancer cell line in the study, with T47D having a distinct expression pattern from MCF‐7 and MDA‐MB‐231.

### 3.3 miRNA‐seq analysis of breast cancer cells upon curcumin treatment

The MCF7, MDA‐MB‐231, and T47D cells were treated with curcumin as mentioned above, and miRNA‐seq was performed. To prepare the miRNA library, three biological replicates were pooled. Sequencing was performed with 2 sets of biological replicates. More than 20 million reads were obtained for each sample. 82%–97% alignment was achieved with processed reads using the reference genome (hg38) (Supplementary Table 2), and differentially expressed (DE) miRNAs (log2 fold change >0.5) in three different human breast cancer cell lines were obtained. 2169 miRNAs were DE for MCF7, 1989 for MDA‐MB‐231, and 2102 for T47D after curcumin treatment. Of these DE miRNAs, 44%, 51%, 61% were upregulated in MCF7, MDA‐MB‐231, T47D, respectively, and 56%, 49%, 39% DE miRNAs were downregulated in MCF7, MDA‐MB‐231, T47D respectively after curcumin treatment (Supplementary Figure 2B).

**FIGURE 3:**
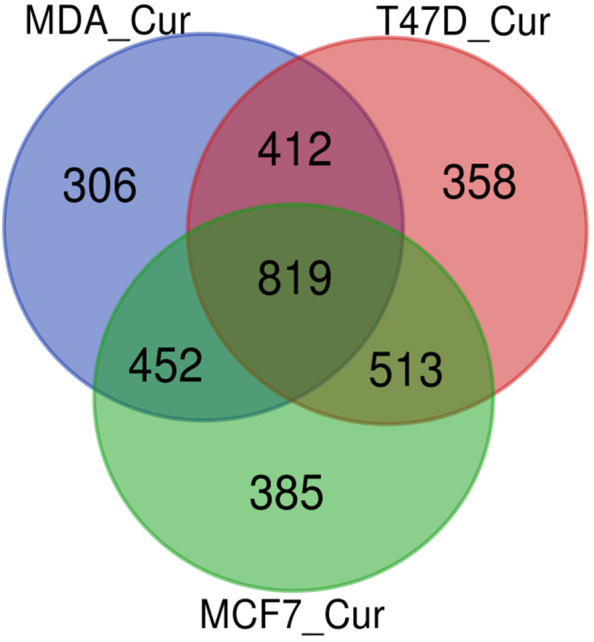
Venn diagram for DE miRNAs from curcumin treated MCF7, MDA‐MB‐231, and T47D cells

819 miRNAs were commonly regulated between all three breast cancer cells (Figure 3). ~4% of miRNAs were commonly regulated among every two cell lines, and ~15–18% of miRNAs were uniquely regulated in each cell line after curcumin treatment. We then analyzed tumor suppressor (TS) and oncogenic miRNA (oncomiR) on the common DE miRNAs. A validated list of 39 TS miRs and 17 oncomiRs specific for breast cancer was literature mined for breast cancer (Supplementary Table 3). The percentage for the common DE miRNAs was calculated for all three breast cancer cells after curcumin treatment (Table 2). Table 3 shows the list of these miRNAs.

**TABLE 2:** Percentage of common DE TS miRNAs and oncomiRs regulated by Curcumin

	MDA‐MB‐231	MCF7	T47D
% Common TS miRNAs upregulated by Curcumin	37.5	32	62.5
% Common oncomiRs downregulated by Curcumin	50	77	28.6

**TABLE 3:** List of TS miRNAs upregulated and oncomiRS downregulated in breast cancer cells upon curcumin treatment

MCF7 OncomiRs downregulated	hsa‐miR‐1207‐5p,hsa‐miR‐210,hsa‐miR‐492,hsa‐miR‐191,hsa‐miR‐374a,hsa‐miR‐155,hsa‐miR‐191‐5p
MCF7 TSmiRs upregulated	hsa‐miR‐22,hsa‐miR‐143,hsa‐miR‐421,hsa‐miR‐30c‐2‐3p,hsa‐miR‐148a,hsa‐miR‐126
MDA OncomiRS downregulated	hsa‐miR‐519a‐3p,hsa‐miR‐210
MDA TSmiRs upregulated	hsa‐miR‐148a,hsa‐miR‐543,hsa‐miR‐494,hsa‐miR‐206,hsa‐miR‐708, hsa‐miR‐512‐5p
T47D OncomiRS downregulated	hsa‐miR‐374a,hsa‐miR‐155
T47D TSmiRs upregulated	hsa‐miR‐140‐5p,hsa‐miR‐204,hsa‐miR‐497,hsa‐miR‐22,hsa‐miR‐204‐5p,hsa‐miR‐335,hsa‐miR‐494,hsa‐miR‐126,hsa‐miR‐206,hsa‐miR‐211‐5p,hsa‐miR‐708,hsa‐miR‐193a,hsa‐miR‐100,hsa‐miR‐424,hsa‐miR‐455

### 3.4 Integrated mRNA–miRNA seq analysis of breast cancer cells upon curcumin treatment

**FIGURE 4:**
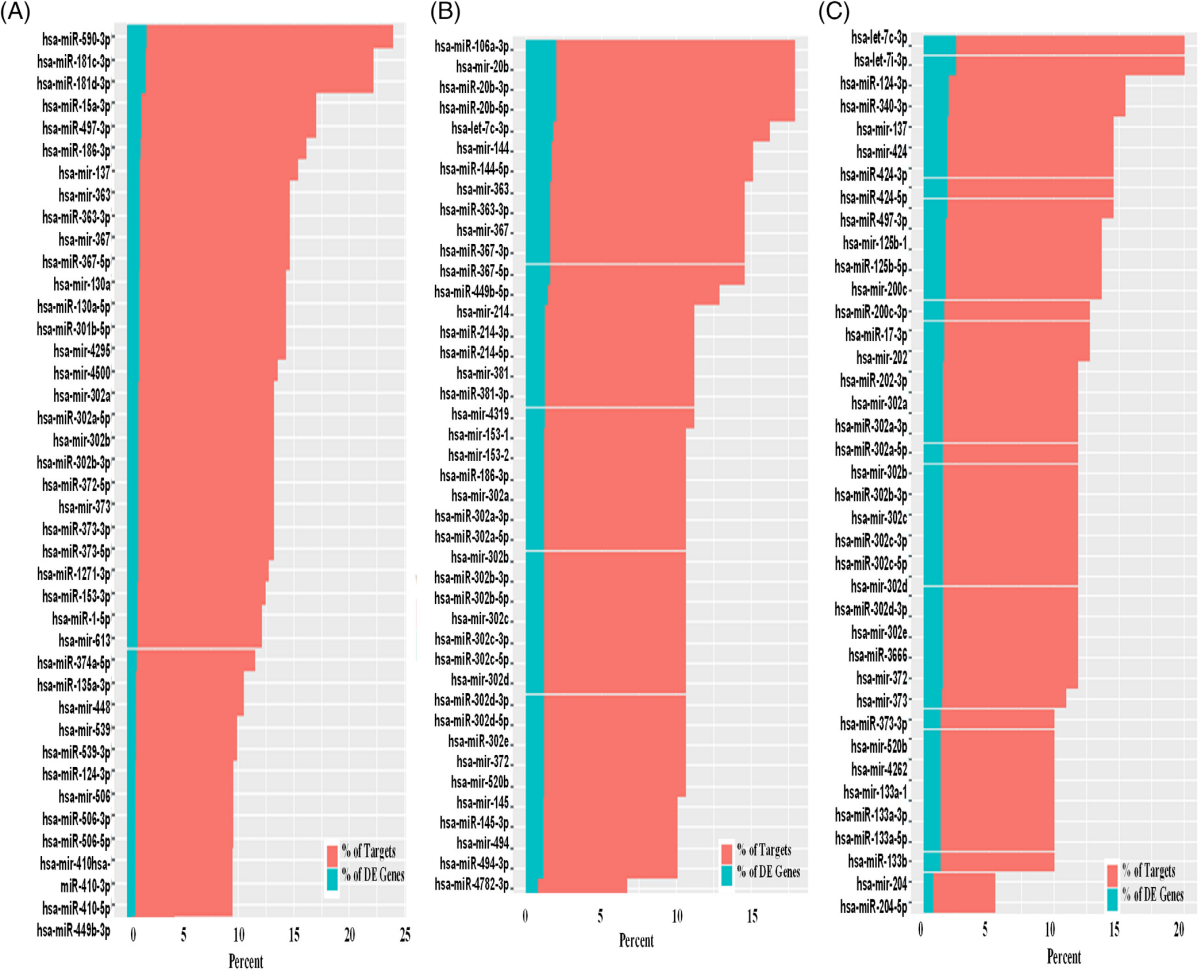
miRmapper output for curcumin treatment on MCF7 (A), MDA‐MB‐231 (B), and T47D (C) cells

The integrated analysis of mRNA–miRNA of breast cancer cell lines namely, MCF7, MDA‐MB‐231, T47D upon curcumin treatment was performed and all the DE miRNAs with log fold change >0.5 and mRNA DE genes with log fold change >1.5, *p*‐value <0.05 were given as input for miRTarVis^40^, ^41^analysis. miRTarVis returned inversely related pairs of miRNA–mRNA, which was used as input for miRmapper.^42^ Using miRmapper, we obtained the list of miRNAs that regulated the maximum number of DE genes. The top 40 miRNAs regulating DE genes from each cell line have been represented as a bar graph (Figure 4). It is known that one miRNA can regulate several genes. Figure 4 shows that 10%–20% of the genes are targets of a particular miRNA, but only 1%–2% of them showed differential expression upon drug treatment. In MCF7 miR‐590‐3p, in MDA‐MB‐231 miR‐106a‐3p and in T47D let‐7c‐3p regulates maximum number of DE genes upon curcumin treatment. miR‐590‐3p is known to regulate the proliferation, apoptosis by targeting PTPN1 via the JNK/STAT/NF‐kB pathway.^55^, ^56^ miR‐106a and let‐7 are also known to regulate the NF‐kB pathway,^57^, ^58^ confirming the previous observations in several cancer cell lines that curcumin exerts its effect via the inhibition of the NF‐kB pathway. To dissect the mechanism of NF‐KB inhibition induced by curcumin treatment, we further analyzed the NF‐kB pathway in all three cells upon curcumin treatment. We identified DE miRNAs whose regulation might influence the NF‐KB pathway. We checked for the downstream targets of NFKB in the DE gene list too. NKFB is a major TF and is known to target 1667 distinct genes.^59^ Out of these, 526, 89, and 52 targets were found DE in the MCF7, MDA‐MB‐231, and T47D gene list, respectively, upon curcumin treatment (Supplementary File 2). Among these, CCL21 was the only common NFKB target among the DE gene list of three cell lines indicating that NF‐KB mediated gene regulation was cell type specific. We also prepared a miRNA–mRNA network for NFKB targets in each cell line (Supplementary Figure 3). The common target CCL21 was regulated by miR‐370, miR‐370‐3p in MCF7 cells.

### 3.5 miRNA–mRNA network analysis

**FIGURE 5:**
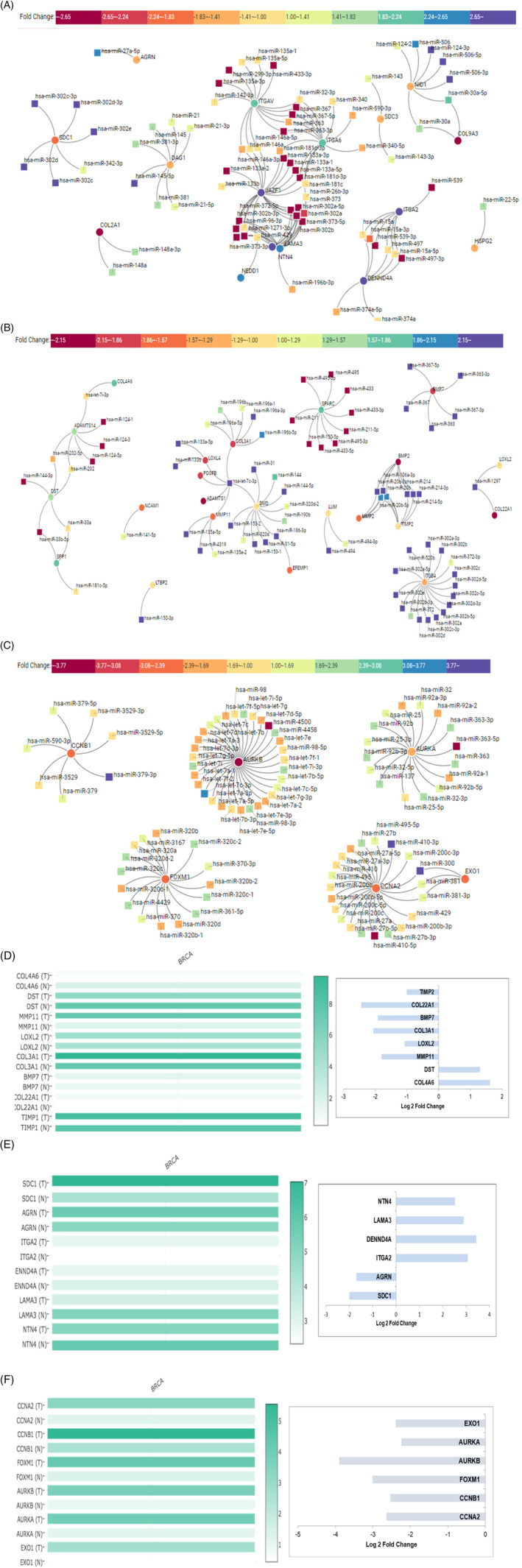
miRNA–mRNA network for non‐integrin and laminin interactions, regulated by curcumin in MCF7 cells (A). miRNA–mRNA network for ECM organization and degradation, regulated by curcumin in MDA‐MB‐231cells (B). miRNA–mRNA network for G2/M transition and G2 cell cycle arrest, regulated by curcumin in T47D cells (C). Drug induced changes, which were either upregulated/downregulated for expression in tumor and reverted to normal expression MDA‐MB‐231 (D), MCF7 (E), and T47D (F)

We built a miRNA–mRNA network for pathways regulated by curcumin in each cell line. Genes enriched in non‐integrin membrane‐ECM interactions and laminin interactions were selected for MCF7; genes enriched in Extracellular matrix organization and degradation were selected for MDA‐MB‐231, and genes enriched in cell cycle arrest and G2/M transition were selected for T47D. Using miRtarvis+, we built an interaction network of miRNA–mRNA (Figure 5A–C). We got an intricate network of miRNA–mRNA for all three cell lines.

The genes enriched in each network were subjected to GEPIA analysis.^60^ Most of the genes in the network restored normal expression (compared to the tumor) after curcumin treatment in each cell line (Figure 5D–F). Integrin and laminin interacting genes like SDC1 and AGRN were downregulated and LAMA3 and NTN4 were significantly upregulated in MCF7 cells upon curcumin treatment. As shown in Figure 5A, miR‐302c, miR‐302d, and miR‐302e regulate SDC1 expression, miR‐27a‐5p regulates AGRNA expression and miR‐196b‐3p regulated NTN4 expression in MCF7 post curcumin treatment. Curcumin significantly downregulated ECM organization and degradation genes like MMP11, LOXL2, and BMP7 in MDA‐MB‐231 cells. miR‐135a‐5p, let‐7c‐3p, miR‐4319, miR‐135a‐2 regulate MMP11 expression, miR‐1297 regulates LOXL2 expression, and miR‐367, miR‐363 regulate BMP7 expression in MDA‐MB‐231 cells upon curcumin treatment (Figure 5B). G2/M transition and G2/M arrest regulating genes like CCNA2, CCNB1, FOXM1, EXO1, AURKA, and AURKB were significantly downregulated in T47D cells upon curcumin treatment. miR‐590‐3p, miR‐379, and miR‐3529 regulated CCNB1 expression, miR‐300, miR‐381 together regulated CCNA2, EXO1 expression, let7 family members were mainly involved in AURKB expression, miR‐302 family regulated FOXM1 expression while miR‐363, miR‐92, miR‐32, and miR‐25 regulated AURKA expression in T47D cells upon curcumin treatment (Figure 5C).

Network analysis showed miR‐363, curcumin commonly regulated miR‐363‐3p in MCF7, MDA‐MB‐231, and T47D. miR‐363 targeted BMP7 in MDA‐MB‐231 cells and regulated the ECM pathway. miR‐363 targeted integrins like ITGAV, ITGA6 in MCF7, and AURKA in T47D cells to regulate the cell cycle G2/M phase. Dolati et al. have reported regulation of miR‐363 by nano curcumin treatment in multiple sclerosis,^61^ indicating miR‐363 as one of the curcumin targets. These are novel miRNA–mRNA pairs of curcumin regulation in breast cancer that need further validation.

## 4 DISCUSSION

In this study, the whole transcriptome (mRNA and miRNA) post curcumin treatment in mesenchymal MDA‐MB‐231 (TNBC), epithelial hormone‐responsive MCF7 (luminal), and T47D (luminal) breast cancer cells have been explored. To evaluate the transcriptomic changes, IC50 values of curcumin on all three cell lines were obtained.^47^ This is the first study identifying differentially expressed genes, miRNAs, and altered pathways by using a very effective integrated miRNA–mRNA approach post curcumin treatment in breast cancer cell lines.

Identification of DE genes and DE miRNAs can be good predictors of response to chemotherapy, biomarker selection, early diagnosis of disease subtypes, and the development of targeted therapeutics.^62^ In addition, cancer heterogeneity and evolution, cancer drug resistance, and the cancer microenvironment can be well studied using transcriptomic data.^63^ Tools like the NCI Transcriptional Pharmacodynamics Workbench (NCI TPW) capture gene expression modulation by molecular pathway, drug target, and association with drug sensitivity across the NCI‐60 panel in response to 15 cytotoxic, targeted anticancer agents.^16^ The understanding of gene regulation involves several molecular players. Integration of the molecular players enhances the knowledge of the regulatory mechanism in complex cellular systems in greater depths, potentially decreases false discovery rate, and facilitates interventional experiments to validate the targets.^64^ In addition, the integration of multi‐omic platforms helps identify relevant miRNA–mRNA pairs that can be putative targets for therapy and diagnostic, prognostic, and predictive markers.^65^

The study showed that 22 DE genes and 819 miRNAs were common in all cell lines after curcumin treatment. More than 48% of genes were uniquely regulated after curcumin treatment. Similarly, RNA‐seq post Shikonin treatment showed more than ~60% DE genes uniquely regulated in MCF7, MDA‐MB‐231, and SK‐BR‐3.^14^ ~15% of miRNAs were uniquely regulated by curcumin in breast cancer cells, indicating miRNA expression to be cell‐type specific, where MCF7 and T47D represent luminal A (ER +ve, PR +ve, HER2 −ve) type whereas MDA‐MB‐231 represent basal, claudin‐low subtype (ER −ve, PR −ve, HER2 −ve),^31^, ^66^

Curcumin regulated Antigen processing and presentation, Interferon alpha/beta/gamma signaling in MCF7, MDA‐MB‐231, whereas pathways related to cell cycle regulation in T47D. In a previous study, Ruihua Li et al. reported that curcumin induced ferroptosis in MCF7, MDA‐MB‐231 cells by upregulating HO‐1 and downregulating GPX4.^67^ The antigen processing and presentation pathways are altered by cancer cells to evade the immune response, which leads to tumor development.^68^ Various chemotherapeutic drugs like Cisplatin,^69^ Gemcitabine,^70^ Melphalan^71^ are used to enhance antigen presentation by the tumor cells, which upregulate MHC‐I, II molecules.^72^ Curcumin upregulates MHC‐I, II molecules which might help sensitize the tumor cells to the cytotoxic T cells (CTLs). In MDA‐MB‐231 cells, curcumin showed regulation of ECM, which plays a significant role in breast metastasis.^73^, ^74^ Naci Cine et al. also reported the role of curcumin in regulating ECM by modulating pathways related to adherens junction.^75^ Overall, curcumin treatment altered cell cycle, apoptosis, and extracellular matrix regulated pathways in breast cancer cells.

The overall tilt in the balance between oncogenic to tumor suppressor after all treatments were evident. Breast cancer progression involves genetic events like an amplification of oncogenes and loss of function of TSGs, leading to malignancy.^76^ Curcumin treatment showed significant downregulation of oncogenes, oncomiRs, and an appreciable increase in TSGs and TSmiRs. For the first time, we showed that oncogenes like FAM83D, SAPCD2 were downregulated upon curcumin treatment in all three cells, and TSGs like ZNF292,^77^ NKAPL, and CCL21 were upregulated in breast cancer cell lines. FAM83D^78^, ^79^, ^80^ and SAPCD2^81^ are known to promote tumorigenesis, induce drug resistance and promote invasiveness. FAM83D promotes cell proliferation and motility by downregulating TSG FBXW7.^48^ Also, FAM83D is a potential biomarker in TNBC.^82^ The promoter of NF‐κB‐Activating Protein‐Like (NKAPL) is hypermethylated in breast^83^, ^84^ and hepatocellular^85^ carcinoma leading to its downregulation, which is correlated, with poor prognosis. CCL21 is a TSG that improved the immunogenicity of MCF7 cells with the assistance of TLR2 and triggered the antitumor response of lymphocytes in vivo.^86^

miR‐21, miR‐27b, miR‐10b‐3p, and miR‐200a‐5p have been reported oncomiRs in breast cancer whereas miR‐26a/b, miR‐628, miR‐205, and miR‐124 have been reported TS‐miRs in breast cancer.^87^ Curcumin downregulated OncomiRsmiR‐21, miR‐210, miR‐155, miR‐374a, and miR‐519a‐3p, and upregulated TS miRNAs like miR‐708, miR‐494, miR‐22, and miR‐148a in breast cancer cells. miR‐374a is reported to promote tumorigenesis in TNBC cells by targeting arrestin beta 1(ARRB1), which has a positive association with TNBC patient survival.^88^ Like curcumin, the derivative of Isoliquiritigenin, a natural flavonoid, represses miR‐374a in MDA‐MB‐231 cells and inhibits cell proliferation, foci formation, migration, invasion. miR‐519a‐3p is another oncomiR that is reported to confer tamoxifen resistance in ER +ve breast cancer.^89^ In addition, miR‐519a‐3p leads to apoptosis resistance and their evasion from immunosurveillance.^90^ Thus, downregulation of miR‐519a‐3p by curcumin can be considered as a positive outcome. miR‐494 inhibits breast cancer progression by targeting PAK1^91^ and inducing apoptosis.^92^ miR‐22, a TS‐miR, inhibits breast cancer metastasis by targeting SIRT1^93^ and inducing cellular senescence.^94^ miR‐148a functions as TS of breast cancer metastasis^95^ via inhibiting migration, invasion by targeting WNT‐1.^96^ In all, curcumin regulated unique miRNA and mRNA in MCF7(luminal), MDA‐MB‐231(TNBC), and T47D (luminal, in a cell type specific manner.

A practical and unique approach was used for mRNA‐miRNA data integration using two tools, miRTarVis^40^, ^41^ and miRmapper.^42^ miRmapper is a tool for the interpretation of miRNA–mRNA interaction networks. It helps us quantify the genes regulated by miRNA in a given miRNA−mRNA seq dataset. miRmapper showed that 15%–20% of the genes are regulated by miRNAs, of which around 4% DE genes were regulated by miRNAs under each treatment. The integrated approach using miRmapper thus helped focus on the predicted targets of differentially expressed miRNAs (DEMs) that were also differentially expressed following drug exposure, indicating miRNA modulation is one of the mechanisms by which curcumin regulates gene expression. Lizarraga et al. also used a miRNA−mRNA integrated approach which showed that benzo[α]pyrene (BaP) is a genotoxic carcinogen modulating apoptotic signaling, cell cycle arrest, DNA damage response, and DNA damage repair pathways.^26^ Another study revealed the lipid metabolism mediated mechanism of Fructus Meliae Toosendan (FMT), a traditional Chinese medicine, to induce liver injury using an integrated miRNA–mRNA approach.^27^

miRmapper analysis showed that in MCF7 miR‐590‐3p, MDA‐MB‐231 miR‐106a‐3p, and T47D, let‐7c‐3p regulates the maximum number of DE genes upon curcumin treatment. All these miRs regulate the NFKB pathway indicating the pivotal role of curcumin in the pathway. Thus, we mined for NFKB targets in the DE gene list and their respective regulating DE miRNAs. We obtained 526 DE NFKB targets in MCF7 cells, 89 in MDA‐MB‐231, and 56 in T47D cells. CCL21 was commonly DE in all the cells after curcumin treatment, indicating its role in immunomodulating breast cancer cells. Curcumin is known to interact with various immunomodulators like dendritic cells, macrophages, both B and T lymphocytes, cytokines, and various transcription factors with their downstream signaling pathways.^97^, ^98^, ^99^ NFKB is a major immunomodulator regulated by curcumin.^100^, ^101^ Our approach showed an intricate miRNA–mRNA network for NFKB regulation via curcumin. miR‐370, miR‐370‐3p that regulates CCL21, has an oncogenic role in breast cancer^102^, ^103^ and was downregulated by curcumin in MCF7 cells.

miRNA−mRNA networks were generated for Non‐integrin membrane‐ECM interactions and Laminin interactions in MCF7, Extracellular matrix organization and its degradation pathways in MDA‐MB‐231 and Cell Cycle Arrest and G2/M transition pathways in T47D. We got intricate networks for these pathways in curcumin‐treated cells. In addition, GEPIA analysis of these genes in breast cancer helped classify them as TSG or oncogene and further interpret the effect of curcumin on breast cancer cells. SDC1 regulates ECM fiber organization in breast cancer stromal fibroblasts and thus is involved in cell motility.^104^ It promotes breast cancer metastasis to the Brain by regulating cytokines.^105^ Our network shows downregulation of SDC1 via miR‐302c, miR‐302d, and miR‐302e in MCF7 cells post curcumin treatment. Guo et al. have shown downregulation of SDC1 via miR‐302a in ovarian cancer.^106^ AGRN is overexpressed in both primary^107^ and highly metastatic tumors.^108^ For the first time, we show downregulation of AGRN in MCF7 cells via miR‐27a‐5p post curcumin treatment. The promoter of LAMA3 is methylated in breast carcinoma, and the frequency of methylation is associated with tumor stage and tumor size,^109^ indicating that LAMA3 upregulation might have a tumor‐suppressive role in breast cancer. NTN4 is a secreted protein and is downregulated in breast cancer. When overexpressed, it leads to the inhibitory effect on invasion, migration via regulation of epithelial‐mesenchymal transition (EMT)‐related biomarkers.^110^ NTN4 upregulation post curcumin treatment via miR‐196b‐3p in MCF7 cells indicates a positive outcome.

Downregulation of MMP11, LOXL2, and BMP7 by curcumin in aggressive, mesenchymal MDA‐MB‐231 cells indicates a positive outcome. miR‐125b is known to regulate MMP11 expression in breast cancer.^111^ Here we show other miRNAs like miR‐135a‐5p, let‐7c‐3p, miR‐4319, and miR‐135a‐2 that regulate MMP11 expression upon curcumin treatment. MMP11 expression by cancer‐associated fibroblasts (CAFs) and intratumoral mononuclear inflammatory cells (MICs) was associated with relapse‐free survival (RFS) and overall survival (OS) in breast cancer.^112^ The lysyl oxidase‐like protein LOXL2 promotes lung metastasis in breast cancer via premetastatic niche formation.^113^ Hence, LOX inhibition is now considered an effective therapeutic approach for breast cancer treatment.^114^ Our study showed that curcumin regulated miR‐1297 to modulate LOXL2 expression. BMP7 is another marker for proliferation, migration, and invasion of breast cancer cells,^115^ and BMP7 protein is detected in breast tumors.^116^ One of the modes of downregulation of BMP7 is via upregulation of miRNA. Curcumin modulated BMP7 downregulation via miR‐367, miR‐363. BMP7 is also known to be regulated by miR‐22 in the kidney.^117^

CCNA2 and CCNB1 are prognostic markers for ER +ve breast cancer and are closely associated with hormone therapy resistance.^118^, ^119^ miR‐219‐5p is known to regulate CCNA2, while miR‐144,^120^ and miR‐718^121^ regulate CCNB1. Here we show other miRNAs that downregulate CCNA2, CCNB2 post curcumin treatment. FOXM1 is a master transcription factor that regulates breast cancer s proliferation, mitosis, and EMT.^122^, ^123^ FOXM1 is overexpressed in breast tumors, including TNBC,^124^ and is strongly associated with tumor size, lymphovascular invasion, lymph node metastases, and advanced metastatic stages.^125^ Targeting FOXM1 can thus be a promising therapeutic strategy to treat resistant, aggressive, metastatic breast cancers.^122^, ^126^ Our study showed that curcumin had the potential to downregulate FOXM1 in T47D cells, which signifies the therapeutic value of curcumin. Curcumin significantly downregulated ECM organization and degradation genes like MMP11, LOXL2, and BMP7. AURKA and AURKB were downregulated upon curcumin treatment and both promote tumorigenesis in both solid and hematological malignancies.^127^ Both are highly expressed in breast cancer and are associated with poor patient survival and worst prognosis.^128^, ^129^ Basal‐line breast cancer exhibits AURKA gene amplification and elevated mRNA expression.^130^ Hence, targeting aurora kinases can provide an effective therapeutic solution for treating breast cancer.^131^ Our study showed that the let7 family might be regulating AURKB in breast cancer, which needs further exploration. Let‐7b‐5p was reported to target AURKB in asthenozoospermia,^132^ although, it is the role needs further validation in cancer. miR‐124‐3p regulates AURKA in bladder cancer.^133^Our study showed other miRs like miR‐363, miR‐92, miR‐32, miR‐25 that regulated AURKA expression in T47D cells upon curcumin treatment.

miR‐363‐3p functions as TS miRNA in lung cancer,^134^, ^135^ colorectal cancer,^136^ papillary thyroid cancer^137^ and glioma.^138^ Curcumin regulating miR‐363 was reported by Dolati et al.^61^ Here we show the first time regulation of miR‐363 and miR‐363‐3p by curcumin in breast cancer cells via the NGS approach. miR‐363 regulated different genes in each cell line leading to the regulation of three distinct pathways.

In conclusion, curcumin regulates miRNA and mRNA in a cell type specific manner. Curcumin altered different pathways in breast cancer cell lines such as cell cycle, migration, invasion, and so forth. The integrative analysis led to the detection of miRNAs and mRNAs pairs, which can be used as biomarkers, associated with carcinogenesis, diagnosis and treatment response in breast cancer.

## CONFLICT OF INTEREST

The authors have stated explicitly that there are no conflicts of interest in connection with this article.

## AUTHOR CONTRIBUTIONS

All authors had full access to the data in the study and take responsibility for the integrity of the data and the accuracy of the data analysis. *Conceptualization*, S.N. and B.C; *Methodology*, S.N., S.D., B.C.; *Investigation*, S.N. and B.C.; *Formal Analysis*, S.N.; *Resources*, B.C.; *Writing ‐ Original Draft*, S.N.; *Writing ‐ Review & Editing*, S.N. and B.C; *Visualization*, S.N. and B.C.; *Supervision*, B.C.; *Funding Acquisition*, B.C. SN and BC conceived the idea, designed the experiments, analyzed the data and wrote the manuscript. SN performed experiments on breast cancer cells. SN and SD performed the bioinformatics analysis. All authors reviewed the manuscript. All authors read and approved the final manuscript.

## ETHICAL STATEMENT

The study was given institutional approval.

## Supporting information


**Supplementary Table 1** Inhouse Sequencing mRNA Data Summary for total reads and overall alignment with Hg38 Reference genome
Supplementary Table 2: Inhouse Sequencing miRNA Data Summary for total reads and overall alignment with Hg38 Reference genome
Supplementary Table 3: List of TS miRs and oncomiRs regulated in breast cancer
Supplementary Table 4: Sequences for RT‐PCR primers used un the study
Supplementary Figure 1: PCA plots for Curcumin treated MCF7,MDA‐MB‐231 and T47D mRNA(A) and miRNA(B) samples.(Abbreviations‐ Curcumin:Cur; VC: Vehicle Control)
Supplementary Figure 2: Transcriptome Summary of % Upregulation and % downregulation of DE significant mRNAs(A) and miRNAs(B) in Curcumin treatment (.(Abbreviations‐ Curcumin:Cur)
Supplementary Figure 3: miRNA‐mRNA network for NFKB targets regulated by Curcumin in MCF7(A), MDA‐MB‐231(B) and T47D(C) cells. (D) Assessment of NFkB in MCF7 breast cancer cells treated with Curcumin: Western blot analysis NFkB protein was done on Curcumin treated MCF7 cell lysates. The experiment was done in duplicates and representative image is shown. Quantification was done (B) and is represented as bar graph of mean +/− SEM. One sample t test and one way ANOVA test was performed and the p value was calculated between control and Curcumin treated groups (*: *p* value < 0.05, **: *p* value < 0.005)

## Data Availability

The data that support the findings of this study are available from the corresponding author upon reasonable request.
