# Supplementary material for: Curcumin alters distinct molecular pathways in breast cancer subtypes revealed by integrated miRNA/mRNA expression analysis
Source: Cancer Rep (Hoboken). 2022 Jan 4;5(10):e1596. doi: 10.1002/cnr2.1596 (PMC9575497; doi:10.1002/cnr2.1596)
Supplement: Supplementary file 1 — Supplementary Table 1 Inhouse Sequencing mRNA Data Summary for total reads and overall alignment with Hg38 Reference genome Supplementary Table 2: Inhouse Sequencing miRNA Data Summary for total reads and overall alignment with Hg38 Reference genome Supplementary Table 3: List of TS miRs and oncomiRs regulated in breast cancer Supplementary Table 4: Sequences for RT‐PCR primers used un the study Supplementary Figure 1: PCA plots for Curcumin treated MCF7,MDA‐MB‐231 and T47D mRNA(A) and miRNA(B) samples.(Abbreviations‐ Curcumin:Cur; VC: Vehicle Control) Supplementary Figure 2: Transcriptome Summary of % Upregulation and % downregulation of DE significant mRNAs(A) and miRNAs(B) in Curcumin treatment (.(Abbreviations‐ Curcumin:Cur) Supplementary Figure 3: miRNA‐mRNA network for NFKB targets regulated by Curcumin in MCF7(A), MDA‐MB‐231(B) and T47D(C) cells. (D) Assessment of NFkB in MCF7 breast cancer cells treated with Curcumin: Western blot analysis NFkB protein was done on Curcumin treated MCF7 cell lysates. The experiment was done in duplicates and representative image is shown. Quantification was done (B) and is represented as bar graph of mean +/− SEM. One sample t test and one way ANOVA test was performed and the p value was calculated between control and Curcumin treated groups (*: p value < 0.05, **: p value < 0.005) [file CNR2-5-e1596-s001.docx]

Supplementary Tables and Supplementary Figures

| **mRNA** | **Total Reads** | **% Alignment** | **RNA integrity Number(RIN)** | **Ratio of all reads aligned**  **to rRNA regions to total**  **uniquely mapped reads**  **(rRNA rate)** |
| --- | --- | --- | --- | --- |
| **MDA-MB-231** | **47335455** | **78.91** | **9.8** | **Negligible** |
| **MDA-MB-231** | **66478647** | **83.34** | **9.9** | **Negligible** |
| **MDA-MB-231_Cur** | **54612179** | **84.52** | **10** | **Negligible** |
| **MDA-MB-231_Cur** | **36031383** | **84.61** | **9** | **Negligible** |
| **MCF7** | **55887233** | **84.01** | **9.2** | **Negligible** |
| **MCF7 Cur** | **32035465** | **86.09** | **9.5** | **Negligible** |
| **MCF7 Cur** | **46263013** | **83.81** | **9** | **Negligible** |
| **T47D** | **25870576** | **80.88** | **9.1** | **Negligible** |
| **T47D** | **45580543** | **85.58** | **9.1** | **Negligible** |
| **T47D Cur** | **22707099** | **89.58** | **9.6** | **Negligible** |
| **T47D Cur** | **42490313** | **84.26** | **9** | **Negligible** |

Supplementary Table 1: Inhouse Sequencing mRNA Data Summary for total reads and overall alignment with Hg38 Reference genome

| **miRNA** | **Total Reads** | **% Alignment** | **RNA integrity Number(RIN)** |
| --- | --- | --- | --- |
| **MDA-MB-231** | **576044256** | **82.25** | **9.5** |
| **MDA-MB-231** | **48990066** | **82.35** | **9.6** |
| **MDA-MB-231_Cur** | **69069139** | **83.19** | **9.6** |
| **MDA-MB-231_Cur** | **50101870** | **81.51** | **9.9** |
| **MCF7** | **68697278** | **89.44** | **10** |
| **MCF7** | **63580633** | **92.08** | **9** |
| **MCF7 Cur** | **53364574** | **83.1** | **9** |
| **MCF7 Cur** | **56379588** | **86.09** | **9.1** |
| **T47D** | **56819311** | **81.54** | **9.5** |
| **T47D** | **64175416** | **82.15** | **9** |
| **T47D Cur** | **60797531** | **84** | **9** |
| **T47D Cur** | **55955641** | **80.39** | **10** |

Supplementary Table 2: Inhouse Sequencing miRNA Data Summary for total reads and overall alignment with Hg38 Reference genome

| TS miRs | hsa-miR-497, hsa-miR-16, hsa-miR-30c-2-3p, hsa-miR-483-3p, hsa-miR-143, hsa-miR-455, hsa-miR-424, hsa-miR-543, hsa-miR-26a, hsa-miR-206, hsa-miR-15a, hsa-miR-30b, hsa-miR-365, hsa-miR-22, hsa-miR-708  hsa-miR-124a, hsa-miR-195, hsa-miR-26b, hsa-miR-148a, hsa-miR-340  hsa-miR-34a, hsa-miR-138, hsa-miR-494, hsa-miR-33b, hsa-miR-421, hsa-miR-193a, hsa-miR-211-5p, hsa-miR-335, hsa-miR-133a, hsa-miR-124, hsa-miR-204-5p, hsa-miR-204, hsa-miR-101, hsa-miR-296-5p , hsa-miR-29b, hsa-miR-140-5p, hsa-miR-126, hsa-miR-100, hsa-miR-512-5p |
| --- | --- |
| OncomiRs | hsa-miR-1207-5p, hsa-miR-492, hsa-miR-135b, hsa-miR-200c, hsa-miR-141, hsa-miR-331, hsa-miR-200b, hsa-miR-122, hsa-miR-374a, hsa-miR-519a-3p, hsa-miR-191-5p, hsa-miR-21, hsa-miR-203, hsa-miR-155, hsa-miR-210, hsa-miR-191, hsa-miR-24 |

Supplementary Table 3: List of TS miRs and oncomiRs regulated in breast cancer

|  | Forward | Reverse |
| --- | --- | --- |
| CTDSP1 | CGCCATCCCTAAGCAGAC | CCACAGGGATGATGAAGTCC |
| ANKRD12 | TTTTGCGAGTTCATTACAGAGC | AATTGTCTTGCATTAAAGCGATC |
| FAM83D | ACGTTGATTGATGGCATCCG | CCTTGGACTGTGGTTTTCGG |
| ZNF292 | AGGTCACTGCTCATGTATAAC | TAGACTTACAGACTGCTGGT |

Supplementary Table 4: Sequences for RT-PCR primers used un the study

B

A

Supplementary Figure 1: PCA plots for Curcumin treated MCF7,MDA-MB-231 and T47D mRNA(A) and miRNA(B) samples.(Abbreviations- Curcumin:Cur; VC: Vehicle Control)

A

B

Supplementary Figure 2: Transcriptome Summary of % Upregulation and % downregulation of DE significant mRNAs(A) and miRNAs(B) in Curcumin treatment (.(Abbreviations- Curcumin:Cur)


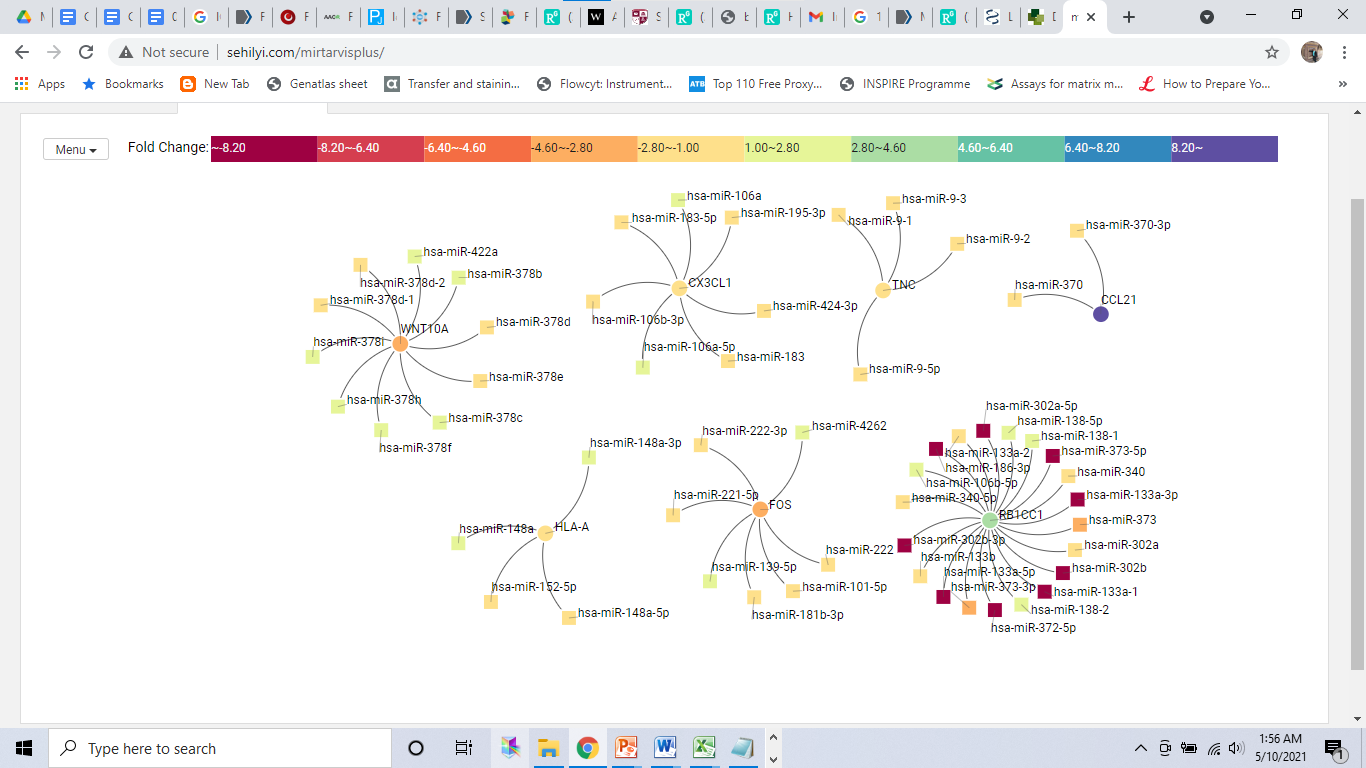


A

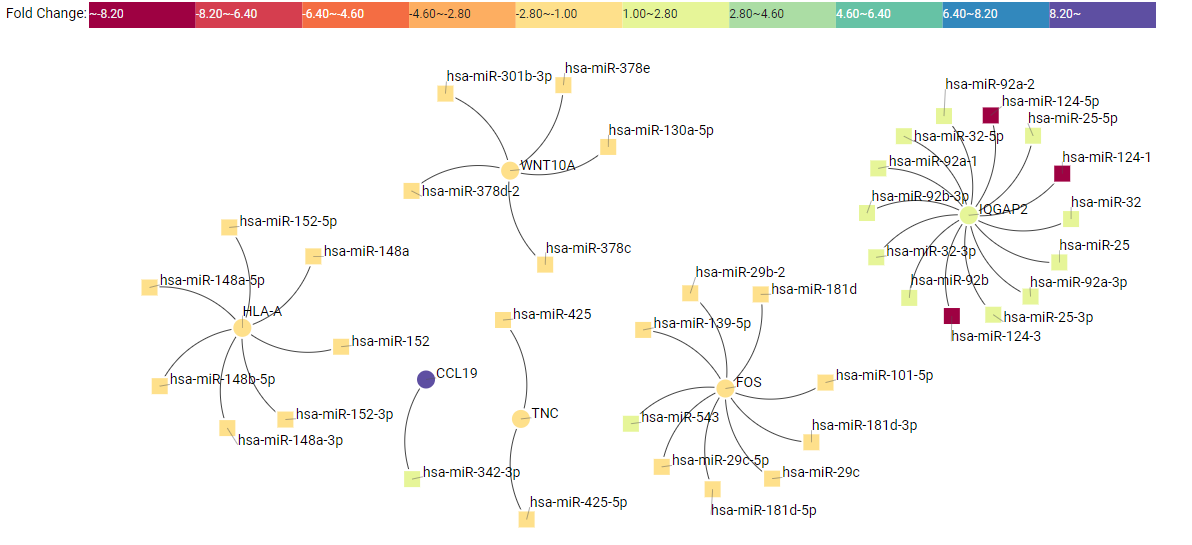


B

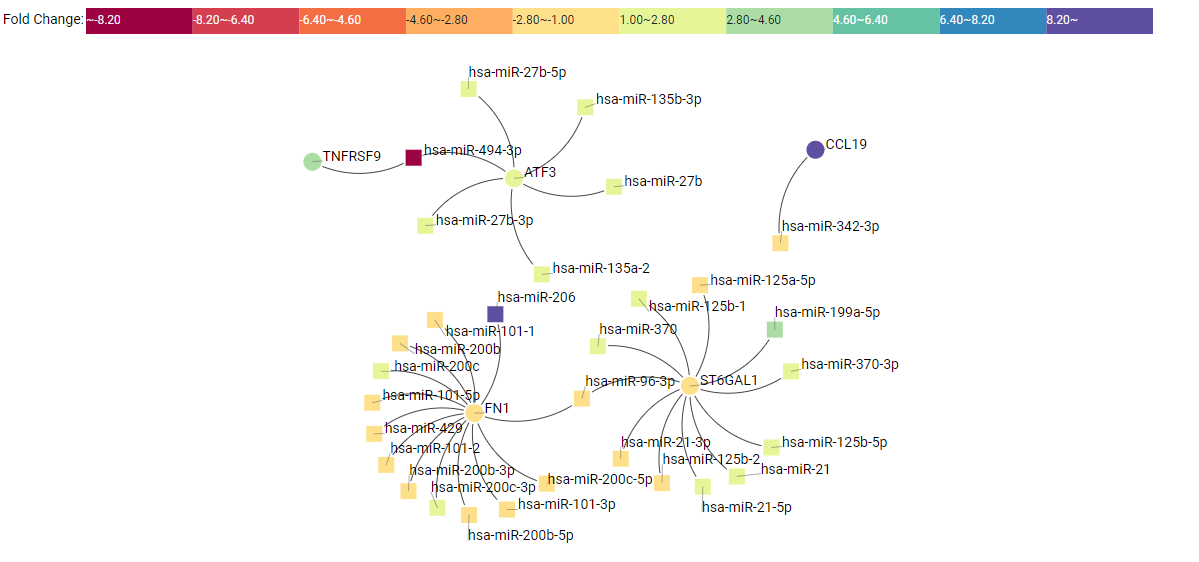


D

C

.

Supplementary Figure 3: miRNA-mRNA network for NFKB targets regulated by Curcumin in MCF7(A), MDA-MB-231(B) and T47D(C) cells. (D) Assessment of NFkB in MCF7 breast cancer cells treated with Curcumin: Western blot analysis NFkB protein was done on Curcumin treated MCF7 cell lysates. The experiment was done in duplicates and representative image is shown. Quantification was done (B) and is represented as bar graph of mean +/- SEM. One sample t test and one way ANOVA test was performed and the p value was calculated between control and Curcumin treated groups (*: *p* value < 0.05, **: *p* value < 0.005)
